# Blockade of Interleukin-6 Trans-signaling in the Presence of Certain Gut Microbiota Induces Mature-onset Obesity in Mice

**DOI:** 10.1016/j.gastha.2025.100819

**Published:** 2025-09-29

**Authors:** Tali Lanton, Dana Eidelshtein, Jacob Rachmilewitz, Rinat Abramovitch, Orit Pappo, Shiran Udi, Saja Baraghithy, Joseph Tam, Sharon Perles, Evan Williams, Sharona Elgavish, Shmuel Ruppo, Hadar Benyamini, Uria Mor, Eran Elinav, Dirk Schmidt-Arras, Ateequr Rehman, Philip Rosenstiel, Anastasios Giannou, Samuel Huber, Stefan Rose-John, Eithan Galun, Jonathan H. Axelrod

**Affiliations:** 1 Goldyne Savad Institute of Gene Therapy, Hadassah Hebrew University Hospital, Jerusalem, Israel; 2 Wohl Institute for Translational Medicine, Hadassah Hebrew University Hospital, Jerusalem, Israel; 3 Department of Pathology, Hadassah Hebrew University Hospital, Jerusalem, Israel; 4 Obesity and Metabolism Laboratory, Institute for Drug Research, School of Pharmacy, Faculty of Medicine, the Hebrew University of Jerusalem, Jerusalem, Israel; 5 Raziel Therapeutics Ltd., Jerusalem, Israel; 6 Luxembourg Centre for Systems Biomedicine, Université du Luxembourg Esch-sur-Alzette, Luxembourg; 7 Info-CORE, Bioinformatics Unit of the I-CORE at the Hebrew University of Jerusalem, Jerusalem, Israel; 8 Immunology Department, Weizmann Institute of Science, Rehovot, Israel; 9 Cancer-Microbiome Research Division, DKFZ, Heidelberg, Germany; 10 Institute of Biochemistry, Christian-Albrechts-University of Kiel, Kiel, Germany; 11 Institute of Clinical Molecular Biology, Christian-Albrechts-University of Kiel, Kiel, Germany; 12 Department of Medicine, University Medical Center Hamburg-Eppendorf, Hamburg, Germany

**Keywords:** Interleukin-6, Sgp130, Obesity, Hepatic steatosis, Metabolic Syndrome

## Abstract

**Background and Aims:**

Interleukin-6 (IL-6) performs multiple roles in regulating metabolic pathways in both mice and man. Here, we examined the age-dependent metabolic phenotype of SGP mice—mice overexpressing sgp130, a factor that specifically blocks IL-6 trans-signaling—that were housed in distant vivaria.

**Methods:**

Transgenic SGP mice engineered to block IL-6 trans-signaling and wild-type littermates were raised in a Jerusalem animal facility to up to 14 months of age and assessed for weight gain, body composition, and metabolic determinants of energy expenditure in young *versus* aged mice. Proteomic and RNA-seq analyses were performed on liver samples as a function of age and genotype.

**Results:**

At ∼6 months of age, weight gain, body fat accumulation, hepatosteatosis, hyperglycemia, and macrophage recruitment to adipose tissue emerged and progressed with age in SGP mice maintained in the Jerusalem animal facility, but not in 3 other vivaria. IL-6/sIL-6R blockade strongly reduced signal transducer and activator of transcription 3 phosphorylation in the liver, and hepatocyte-targeted ablation of signal transducer and activator of transcription 3 recapitulated the IL-6 trans-signaling blockade phenotype. Multiomics analyses of mouse livers revealed age- and genotype-related changes in gene expression profiles attributable to bacterial byproducts. Depletion of the gut microbiota by antibiotic treatment from the age of 6 months reversed the obese phenotype in transgenic mice, confirming the crucial role of the microbiome in the phenotype. Accordingly, the microbiome of mice from the Jerusalem animal facility differed significantly from that of mice from animal facilities in Kiel and Hamburg, Germany, where the same mice did not develop a metabolic phenotype.

**Conclusion:**

These findings reveal the crucial functions of IL-6 trans-signaling in preventing mature-onset body fat accumulation induced by certain intestinal microbiota.

## Graphical abstract

**Figure:**
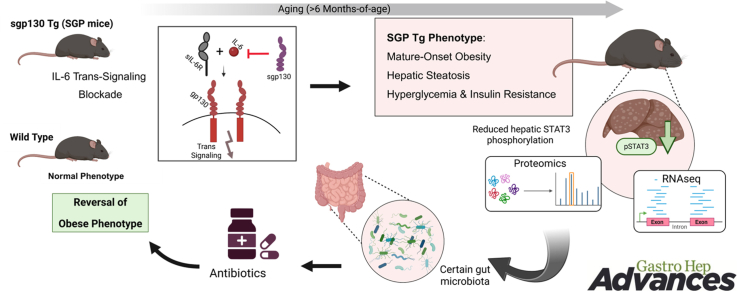

## Introduction

Accumulating evidence indicates that interleukin-6 (IL-6) plays numerous roles in the maintenance of metabolic homeostasis. For example, selective disruption of IL-6 signaling in the mouse liver by targeted ablation of the IL-6 receptor (IL-6R) in hepatocytes increases inflammation and reduces insulin sensitivity.^1^ Similarly, circulating IL-6, which can increase up to 100-fold following physical exercise,^2^ enhances insulin-stimulated glucose disposal and increases insulin sensitivity in peripheral tissues.^3^ In the central nervous system, IL-6-dependent pathways that affect body weight control are exerted in the lateral parabrachial nucleus^4^ and hypothalamus, where they contribute to reduce feeding and maintain peripheral glucose tolerance, especially in the face of leptin and insulin resistance in obesity.^5^, ^6^, ^7^, ^8^ Importantly, IL-6 *knockout* mice have been reported to develop mature-onset obesity, characterized by increased adiposity and hepatic steatosis accompanied by hyperglycemia, hyperinsulinemia, and systemic insulin resistance;^9^^,^^10^ although, one study could not confirm this phenotype despite using genetically identical mice.^11^ Cabonaro *et. al*. reported that functional IL-6-IL-6R-gp130 signaling is required to protect human hepatocytes against excessive lipid droplet accumulation in a humanized murine liver model.^12^ In addition, the long-term blockade of IL-6 signaling by ACTEMRA® (tocilizumab), a recombinant humanized anti-IL-6R antibody, is associated with body weight gain, increased serum lipid and cholesterol levels, and abolishment of exercise-induced reduction in visceral adipose tissue mass in some human patients.^13^^,^^14^ Thus, as noted by Janson and Palsdottir,^15^ IL-6 acts at both peripheral and central sites in the body, where it generally appears to promote metabolic homeostasis.

IL-6 signals via 2 membrane-bound coreceptors, a cognate α-receptor that binds IL-6 (IL-6R) and gp130, a β-receptor that binds the IL-6-IL-6R complex and initiates signal transduction. IL-6R is also notably expressed in a soluble form (sIL-6R), leading to multiple signaling configurations. In its *classical* signaling configuration, IL-6R, which is expressed in limited cell populations, combines with IL-6 to engage ubiquitously expressed gp130 and initiate downstream signaling cascades.^16^ However, sIL-6R, which is normally present in high serum concentrations, can also bind IL-6 and trigger gp130-mediated signaling in a mechanism called *trans-*signaling, including in cells that do not express IL-6R.^17^^,^^18^ Importantly, gp130 is also expressed in a truncated soluble form (sgp130) that is normally present at high levels in the serum, where it can bind and neutralize IL-6-sIL-6R complexes, but not either protein alone, thus acting as a specific antagonist of IL-6 trans-signaling.^19^^,^^20^ IL-6, which is found in the pg/ml range in the blood of unchallenged individuals, can increase several hundred- or thousand-fold during inflammation or infection. In this situation, IL-6 binds to sIL-6R, and the IL-6-sIL-6R complex is neutralized by sgp130. Therefore, sIL-6R and sgp130 act together as buffers for IL-6 in blood.^21^ Peripheral IL-6 trans-signaling blockade can be imposed experimentally by ectopic expression of an sgp130Fc protein, which has also been engineered into the transgenic mouse model—SGP mice—used in this study .^20^^,^^22^

Clinical evidence suggests that peripheral IL-6 trans-signaling may contribute to the maintenance of metabolic homeostasis. For example, elevated serum sgp130 levels have been found to be associated with obesity, diabetes, and insulin resistance in adult patients,^23^, ^24^, ^25^ but not in obese children or adolescents.^26^ Circulating sgp130 levels have also been found to be increased in people with steatotic liver disease and steatohepatitis (metabolic dysfunction–associated steatotic liver disease (MASLD)/metabolic dysfunction–associated steatohepatitis (MASH)), and further increased in diabetes concurrent with steatohepatitis, with clear correlations between HbA_1c_ and sgp130 levels in obesity.^27^ Elevated serum sgp130 levels have also been linked to diabetes and increased body mass index in older individuals who are at a high risk for coronary artery disease and carry the G148C gp130 polymorphism.^28^ This polymorphism has been shown to impair gp130 function,^29^ suggesting that reduced gp130 activity may also pose a potential predisposition to comorbidities of metabolic syndrome. By contrast, Kraakman *et. al.*^30^ reported that IL-6 trans-signaling blockade in young SGP mice did not significantly alter body mass, glucose tolerance, or energy expenditure in comparison with wild-type (WT) controls, and prevented high-fat diet-induced adipose tissue macrophage recruitment without exacerbating insulin resistance. Moreover, blocking IL-6 trans-signaling and alleviating ER stress in the MUP-uPA mouse model efficiently reversed MASH and reduced MASH-driven hepatocellular carcinoma.^31^ Thus, while classical IL-6 signaling appears to be closely linked to metabolic diseases and MASLD, the association between IL-6 trans-signaling and MASLD or MASH remains unclear, and the underlying mechanisms remain unknown. Together, these observations suggest that the inhibition of peripheral IL-6 trans-signaling may be crucial for metabolic homeostasis; however, aging and environmental factors may be critical in its presentation. To test this hypothesis and unfold its potential mechanism(s), we followed SGP mice and WT littermates in the Jerusalem animal facility from early to late ages, and characterized them for metabolic and inflammatory functions in comparison with identical genotypes in other vivaria.

## Materials and Methods

### Animal Care

Mice were maintained in an animal facility at a temperature of ∼23°C in a 12-hour light-dark cycle, under SPF conditions, and received sterile commercial rodent chow (Teklad Global 2918) and water *ad libitum*. Mice were housed in Tecniplast Conventional cages with Teklad Laboratory Grade Sani-Chips bedding (Cat. No. 7090) and enrichment tubes. Maintenance of mice and all experimental procedures were approved by and performed in accordance with the Institutional Animal Care and Use Committee-approved animal treatment protocols (license number OPRR-A01-5011). All animal related experiments adhere to standards articulated in the Animal Research: Reporting of In Vivo Experiments (https://arriveguidelines.org/) guidelines.^32^

### Genetic Mouse Models

*SGP*^+/+^ (C57BL/6N) mice^22^ were crossed with WT (C57BL/6J) mice purchased from Harlan Laboratories (Jerusalem, Israel) to generate heterozygous *SGP*^+/−^ mice that were crossed to generate both homozygous *SGP*^+/+^ mice and WT littermates. Floxed-STAT3 (Stat3^floxP^) (C57BL/6) mice^33^ (kindly provided by E. Razin, Hebrew University of Jerusalem, Israel) were crossed with Alb-Cre^+/+^ (C57BL/6) mice^34^ (kindly provided by D. Wallach, Weizmann Inst. of Science, Rehovot, Israel) to form Stat3^floxP/+^Alb-Cre^+/−^ mice, the offspring of which were then crossed to generate the Stat3^floxP^Alb-Cre (Stat3^ΔHep^) and Stat3^floxP^ strains. See Supplementary Information for further details on the generation and validation of genetic mouse models.

### Study Design

Male mice were used in all experimental groups and were housed in groups consisting of 2–3 mice per cage and fed standard mouse chow *ad libitum*. *Sgp130 mice* and WT littermates were raised to the age of 14 months. Upon sacrifice, liver and adipose tissue samples were removed, snap-frozen in liquid nitrogen for protein and RNA extraction, embedded in frozen tissue sections (OCT), or fixed in 4 percent formaldehyde, and paraffin-embedded for histological and immunostaining analyses. For antibiotic treatment, *SGP* and WT littermate mice raised to the age of 6 months were administered with drinking water containing neomycin (Invivogene), meropenem (Anfarm), and vancomycin (Mylan) at a final concentrations of 0.5 mg/ml (meropenem and vancomycin) or 1 mg/ml (neomycin) in tap water. The control group received tap water only. Antibiotic treatment or control tap water was refreshed twice a week for 3 months, starting at the age of 6 months. Blood sugar levels were measured using a glucometer (Accu-Chek) and test strips (Accu-Chek) at 8 months of age after 16 hours of overnight fasting.

See Supplementary Information for full details on the analyses by magnetic resonance imaging (MRI), metabolic cages, glucose and insulin tolerance testing, insulin secretion, insulin signaling, Western blot, histochemical and immunostaining, flow cytometry, RNA, qPCR, RNA-seq, Sequential Window Acquisition of all Theoretical Mass Spectra (SWATH-MS) proteomics, and taxonomic microbiota analysis.

### Statistical Analysis

Data were evaluated for significance using a 2-tailed Student's *t*-test or Mann–Whitney U test unless otherwise noted. *P* ≤ .05 was considered significant for all analyses. Calculations were performed using GraphPad Prism 6.02 software (GraphPad Software, Inc., San Diego, CA, USA).

## Results

### IL-6 Trans-signaling Blockade Induces Mature-onset Obesity on a Normal Chow Diet

**Figure 1:**
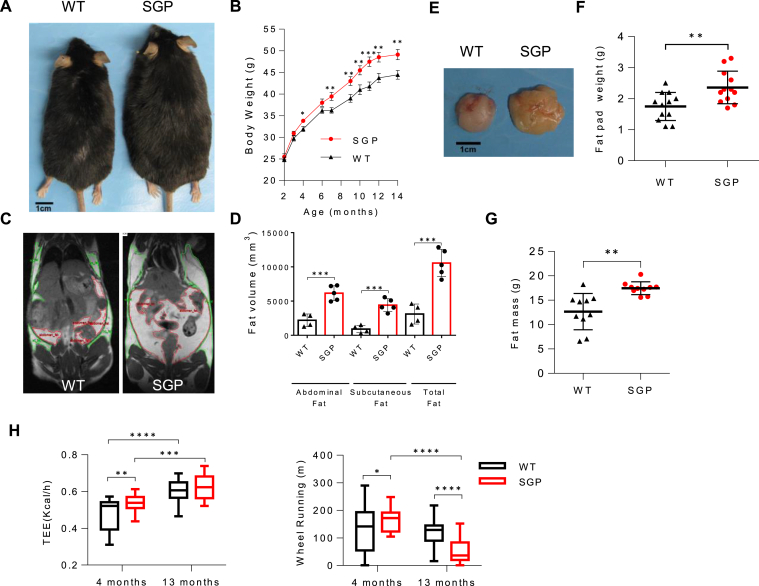
IL-6 trans-signaling blockade induces mature-onset obesity in mice on a normal chow diet. (A) Photograph showing representative male, SGP and WT mice at 14 months of age. (B) Body weight progression from 2 to 14 months of age in male SGP and WT mice (n = 22–25; total = 47). No mice were excluded. The primary observation was repeated in 4 independent experiments in male littermates, and once in both male and female nonlittermate mice. (C) Representative coronal MRI images of SGP and WT mice at 10 months. (D) Quantification of subcutaneous and abdominal fat tissue in mice from MRI images (n = 4–5) (E) Photographic image of representative gonadal fat pads, (F) Quantification of fat pad mass (right) at 14 months (n = 12). (G) Quantification of fat mass at 14 months by EchoMRI (n = 9–10). (H) Metabolic cage assessment of dark cycle TEE and voluntary physical activity (wheel running) at 4 and 13 months of age (n = 12). Data are presented as mean ± standard deviation (SD) (E-F) or mean ± standard error mean (SEM) (B). ∗*P* < .05, ∗∗*P* < .01, ∗∗∗*P* < .001, and∗∗∗∗*P* < .0001, by 2-tailed Student’s t test (B, D, F, G) or by 2-way ANOVA (H). (See also Figures S1 and S2).

At birth, SGP mice (Figure S1A) were indistinguishable from their WT littermates and remained so until approximately 5–6 months of age when body weights began to diverge, with SGP mice becoming visibly and significantly overweight from the age of 7–8 months (Figure 1A and B). Mature SGP mice fed a chow diet displayed increased body fat and decreased lean body mass relative to their WT littermates, as shown by MRI (Figure 1C and D), epididymal fat pad weights (Figure 1E and F), and echoMRI (Figures 1G and S1B). The body weight increase in the mice was largely due to fat accumulation, which, as measured by MRI analysis, increased by approximately 40 percent compared to that in WT mice at 14 months, with roughly equal distribution between subcutaneous and abdominal fat mass (Figure 1D). Metabolic cage analyses provided further insights into the metabolic phenotypes of SGP mice. At 4 months of age, despite similar body composition, SGP mice exhibited a significant increase in total energy expenditure (TEE) compared to WT littermates (Figures 1H and S1C and D). This increase in TEE was not driven by elevated food intake as no significant differences in caloric consumption were observed between the groups. Interestingly, elevated energy expenditure in SGP mice was coupled with increased physical activity, including higher levels of voluntary wheel running, suggesting that these mice were more metabolically active and efficient in energy utilization at an early age. However, this increase in energy expenditure was not sustained. By 13 months of age, the increase in TEE observed at 4 months was diminished, with no significant differences between SGP and WT mice at this later stage. These findings highlight a distinct metabolic shift in SGP mice, which is initially characterized by enhanced energy expenditure and activity; these traits are progressively lost with age. This metabolic shift may contribute to the later onset of excessive fat accumulation and reduced lean mass in aged SGP mice.

**Figure 2:**
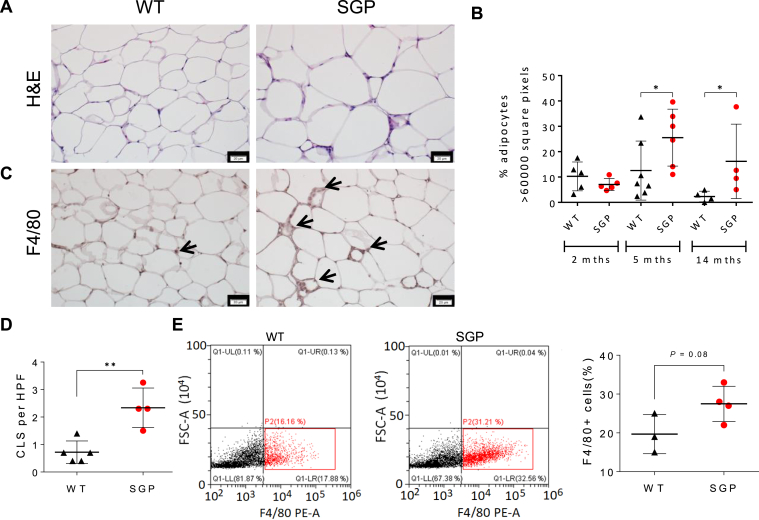
IL-6 trans-signaling blockade induces adipocyte hypertrophy and ATM recruitment. (A) H&E-stained adipose tissue sections from SGP mice and WT littermates at 14 months of age. Scale bars, 20 μm. (B) Quantification of adipocytes with a cell cross-sectional area >6000 square pixels using ImageJ (n = 4–7). (C) Photomicrograph of F4/80 immunostaining of adipose tissue thin sections showing ATMs (red) in CLS (arrows) at 14 months. Scale bars, 20 μm. (D) Quantification of F4/80+ CLS per HPF in adipose tissue thin sections (c) (n = 4). (E) Representative flow cytometry plots (left) and quantification (right) of F4/80-positive cells in the adipose tissue of 11-month-old mice (n = 3–4). F4/80+ cells were quantified in the bottom-right quadrant (gate P2) (red). Data are represented as mean ± SD. ∗*P* < .05, and ∗∗*P* < .01 by 2-tailed, Mann–Whitney test (B) or 2-tailed, Student’s t test (D, E). (See also Figure S2). ATM, adipose tissue-associated macrophage; CLS, crown-like structure; FSC-A, forward scatter area; H&E, hematoxylin and eosin; HPF, high-power field.

**Figure 3:**
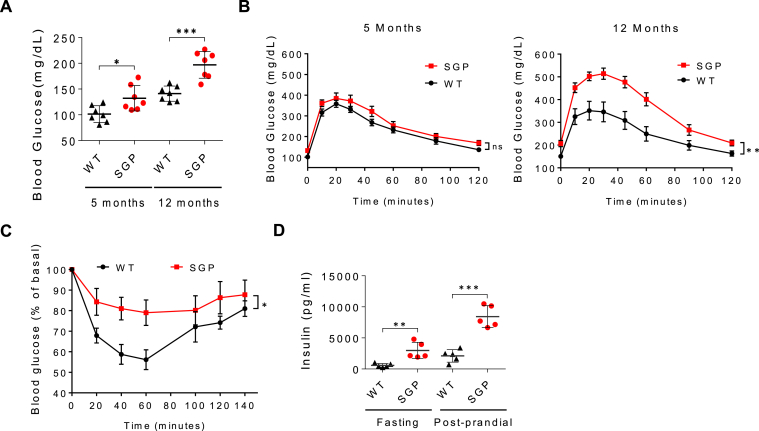
Inhibition of IL-6 trans-signaling induces glucose intolerance and peripheral insulin resistance. (A) Fasting glucose levels in SGP and WT littermates (n = 7). (B) Glucose levels before and after glucose challenge in SGP versus WT mice at 5 (left) and 12 (right) months of age (n = 7). (C) Blood glucose levels in fasting 12-month-old SGP and WT mice at indicated times following insulin injection (i.p.) (n = 7). (D) Fasting and postprandial serum insulin levels in SGP and WT littermates at 13 months of age (n = 5). Data are presented as mean ± SD (A, C, D–G) or mean ± SEM (B, C). ∗*P* < .05, ∗∗*P* < .01, and ∗∗∗*P* < .001 by 2-tailed Student’s *t* test. (B, C) ∗*P* < .05, ∗∗*P* < .01, for comparisons of area under the curve using 2-tailed Student’s *t* test. (See also Figure S3).

Adiposity in SGP mice appeared largely due to adipocyte hypertrophy marked by an increased mean adipocyte cross-sectional area in H&E-stained white adipose tissue biopsies that emerged concurrently with the onset of body weight divergence (Figures 2A and B, and S2). This is in contrast to that reported for young mice of identical genotypes maintained in an animal facility in Australia.^30^ Adipose tissue-associated macrophage recruitment within crown-like structures in adipose tissue, a characteristic of obesity,^35^ as determined by immunostaining and confirmed by flow cytometry analysis, was also increased in SGP mice compared to controls (Figure 2C–E). Together with their weight gain, SGP mice also displayed fasting hyperglycemia and glucose intolerance that worsened with age and both fasting and postprandial hyperinsulinemia and insulin resistance (Figure 3A–D). Western blot analysis showed a strong reduction in ribosomal protein S6 phosphorylation in peripheral tissues (liver, muscle, and adipose) in response to injected insulin, consistent with the presence of peripheral insulin resistance (Figure S3A–C). These changes were also accompanied by increases in fat oxidation and significantly reduced utilization of carbohydrate metabolism in older SGP mice, in line with their hyperglycemia and insulin resistance (Figure S3D).

### The Liver is a Primary Target of IL-6 Trans-signaling in the Prevention of Fat Accumulation

**Figure 4:**
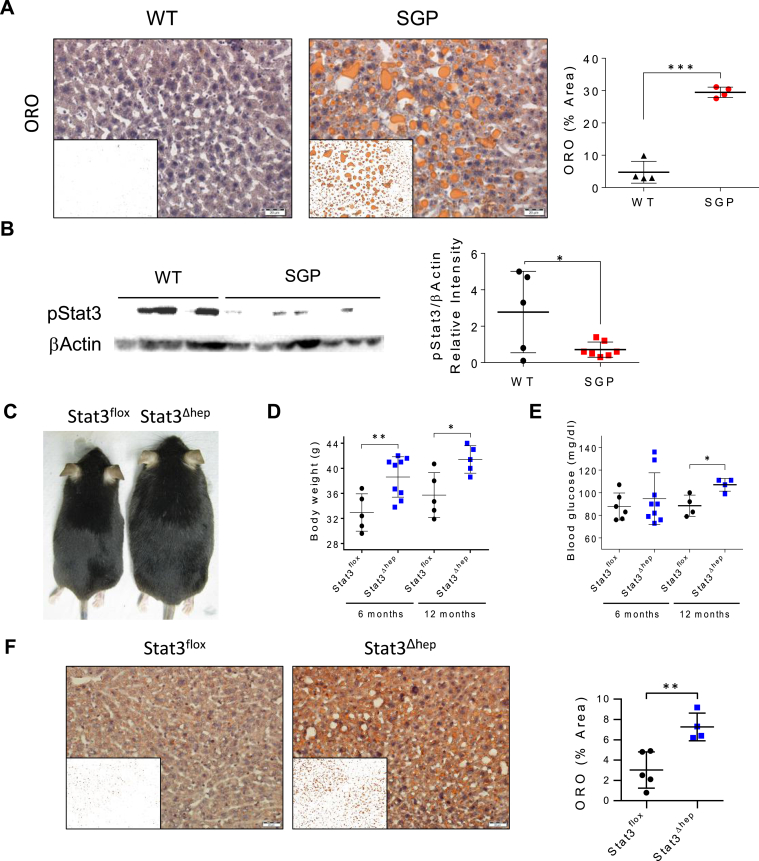
STAT3 signaling in hepatocytes is required for IL-6 trans-signaling-mediated prevention of fat accumulation and hepatosteatosis. (A) Representative images showing hepatic steatosis by ORO staining of livers from SGP and WT littermates at the age of 14 months and ImageJ processed image (inset). Scale bars represent 20 μm. (below) Quantification of the ORO-stained area was performed using ImageJ (n = 4). (B) Phosphorylated Stat3 and β-actin protein levels by Western Blot analysis of liver samples from 5-month-old SGP and WT littermates and quantification of band intensities (right) (n = 5–7). (C) Photographic image showing representative Stat3^Δhep^ and Stat3^floxP^ mice aged 12 months. (D) Body weights of Stat3^Δhep^ and Stat3^floxP^ mice aged 6 and 12 months (n = 5–9). (E) Fasting blood glucose levels in 6 and 12-month-old Stat3^Δhep^ and Stat3^floxP^ mice (n = 4–9). (F) Representative ORO-stained liver sections from Stat3^Δhep^ and Stat3^floxP^ mice at 12 months of age with ImageJ processed image (inset), and quantification of ORO-stained areas by ImageJ (right) (n = 4–5). Scale bars, 20 μm. Data are presented as mean ± SD. ∗*P* < .05, ∗∗*P* < .01, ∗∗∗*P* < .001 by 2-tailed, Student’s *t* test. (See also Figures S4 and S5). ORO, Oil red O.

Hepatic steatosis, as assessed by Oil red O staining in liver biopsies, began to emerge in SGP mice at the Jerusalem facility at 5 months of age, with frank steatosis involving strikingly larger fat vacuoles becoming evident at 14 months (Figures 4A and S4). Interestingly, this is different from previous observations in which treatment of MUP-uPA/SGP mice with BGP-15 reversed hepatic steatosis at the age of 10 months.^31^ We did not detect an increase in inflammation associated with hepatic steatosis. In contrast, the levels of liver Cd11b^+^ monocytes, Kupffer cells and infiltrating macrophages (F4/80), assessed by flow cytometry and immunostaining, appeared either unchanged or reduced in SGP mice compared to their WT littermates (Figure S5A–C). Likewise, we observed no significant changes in the levels of hepatic mRNAs encoding *Cd11b, F4/80, Cd68,* and *Tlr4,* or in the expression of the inflammatory cytokines, *Tnfα, Il-10* and *Il-6* (Figure S5D). Nevertheless, we observed a striking decrease in the levels of phosphorylated signal transducer and activator of transcription 3 (STAT3), a key mediator of IL-6 signaling, in the livers of SGP mice compared to those in their WT littermates (Figure 4B), suggesting that Stat3 activation induced by IL-6-sIL-6R signaling is crucial for the maintenance of metabolic equilibrium in mature WT mice. Importantly, hepatocyte-targeted STAT3 ablation (Stat3^ΔHep^) in mice, generated by crossing Stat3^floxP^ mice with Alb-Cre^+/+^ mice,^33^^,^^34^ induced a metabolic phenotype similar to that of SGP mice, with weight gain and hepatic steatosis emerging from the age of about 6 months compared to Stat3^floxP^ controls, together with hyperglycemia at 12 months of age (Figure 4C–F); thus, largely confirming the previous observations of Inoue *et.al.*^36^ Together, these findings demonstrate that in animals housed in the Jerusalem animal facility trans-signaling blockade reproduces much of the age-dependent metabolic phenotype previously observed in both IL-6 *knockout* mice^9^^,^^10^ and Stat3^Δhep^ mice,^36^ indicating that the liver is a primary target of IL-6 trans-signaling that prevents mature-onset obesity.

### Multiomics Analysis Hints at Age-dependent Crosstalk in Liver with Microbiota

**Figure 5:**
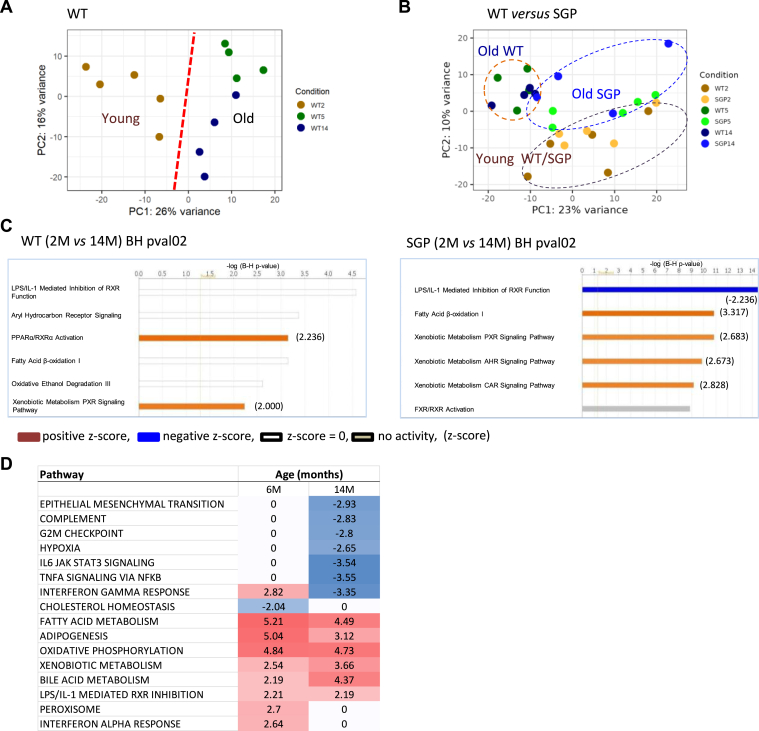
Proteomics and RNAseq analyses reveal age- and strain-dependent changes in gene expression profiles in the livers of WT and SGP mice. (A) PCA of liver proteomes distinguished (red dashed line) between gene expression profiles in younger (2 months) and older (5 and 14 months) WT mice along the PC1 axis. (B) PCA plots of liver proteomes of WT *versus* SGP mice at 2, 5, and 14 months of age clustered old WT mice separately from young (2M) mice. Young WT and SGP groups cluster together, but older SGP mice (5 and 14 months) appear dispersed between and more closely related to young mice on the PC1 axis. (C) The top 6 enriched QIAGEN IPA® Canonical Pathways were enriched by aging (2 months *versus* 14 months) in the liver proteomes of the WT (left) and SGP (right) mice. [-log(B-H *P* value) > 1.3] (*x-axis*) was considered statistically significant. |QIAGEN IPA® Z-scores|(values) > 2 were considered statistically significantly upregulated or downregulated for positive and negative values, respectively. (n = 5 and n = 4 for mice aged 2 and 14 months, respectively). (D) GSEA^38^ -generated heatmap from RNA-seq analysis showed that the top **Pathway** (MSigDB Hallmark gene sets^38^) was significantly enriched (false discovery rate < 0.0001) in the livers of SGP versus WT mice at 6 and 14 months of age. Gene sets enriched in upregulated genes are represented by positive (red) NES values, whereas those enriched in downregulated genes are represented by negative (blue) NES values with a gradual color gradient. Mice at 6 (n = 3) and 14 months (n = 2). (See also Figure S6). GSEA, gene set enrichment analysis; IPA, Ingenuity Pathway Analysis; PCA, principal component analysis.

To reveal age-affected mechanisms in the liver that may potentially underlie fat accumulation in SGP mice, we first compared changes in protein expression patterns in the liver according to age using SWATH-MS proteomics^37^ with bioinformatics analysis. SWATH-MS proteomic analysis of liver samples from WT and SGP mice at the ages of 2, 5, and 14 months identified and quantified 21,927 unique peptides corresponding to 2835 unique protein groups. Surprisingly, although older WT mice were not phenotypically different in adiposity from their young siblings (see Figure 2B), principal component analysis clearly distinguished between younger (2 months) and older (5 and 14 months) mice according to their liver proteomes (Figure 5A). This indicated the presence of inherent age-dependent processes in the livers of the WT mice. A similar Principal component analysis comparison encompassing the proteomes of both WT and SGP mice indicated that the younger mice (2 months old) of both genotypes clustered together, mirroring their phenotypic similarity (Figure 5B). Older (5 and 14 months) WT mice also clustered together and diverged from younger mice of both genotypes. Interestingly, the proteomes of the older SGP mice (SGP5 and SGP14) also appeared to diverge from those of the young mice but remained largely distinct from their older WT (WT5 and WT14) siblings (Figure 5B).

Bioinformatics comparisons using QIAGEN Ingenuity Pathway Analysis (IPA®) of the proteomes of young (2M) *versus* old (14M) mice according to genotype identified several canonical and toxicity-related pathways that were strongly affected by age and appeared common to both genotypes. These included pathways related to fatty acid metabolism (*PPARα/RXRα Activation* and *Fatty acid β-oxidation I*) and metabolism of microbiota byproducts (*i.e. Xenobiotic Metabolism* and *LPS/IL-1 Mediated Inhibition of RXR Function*) that are predicted to be upregulated or downregulated in older mice (QIAGEN IPA® z-score > 2) (Figure 5C). Interestingly, the respective activation or inhibition of these pathways appeared to be strengthened in SGP mice. Comparisons of gene expression patterns in the livers of *SGP versus* WT mice (*ie* between genotypes) using RNAseq and gene set enrichment analysis^38^ showed a significant enrichment (false discovery rate<0.0001) of upregulated genes in the gene sets of both adipogenesis and fatty acid metabolism at both 6 and 14 months of age in the livers of SGP mice (positive Normalized Enrichment Score (NES)), as identified earlier by direct measurements in metabolic cages (Figure S3), as well as a significant enrichment (false discovery rate<0.0001) in downregulated genes of *IL-6 JAK STAT3 signaling* gene set (negative NES) (Figures 5D and S6). Importantly, gene set enrichment analysis^38^ also revealed an enrichment of upregulated genes in liver gene sets related to *xenobiotic metabolism, bile acid metabolism, and LPS/IL-1 mediated RXR inhibition* when comparing the transcriptome of SGP mice to WT siblings (positive NES) (Figures 5D and S6), indicating the influence of microbiota-related by-products on liver gene expression patterns.^39^ Previous studies have indicated that the intestinal microbiota can extensively modulate hepatic gene expression and function in mice without direct contact with the liver, particularly by altering its xenobiotic response to drugs and affecting genes of metabolic function.^39^ Thus, multiomics analysis revealed age-dependent microbiota-related changes in gene expression patterns in the liver that increase in IL-6 trans-signaling-deficient mice. These findings suggest that age-dependent processes leading to body fat accumulation are intrinsic to mice of both genotypes, but that IL-6 trans-signaling in the livers of WT mice counteracts their influence on metabolic equilibrium and masks their presence.

### Mature-onset Obesity in SGP Mice Requires a Gut Microbiota

**Figure 6:**
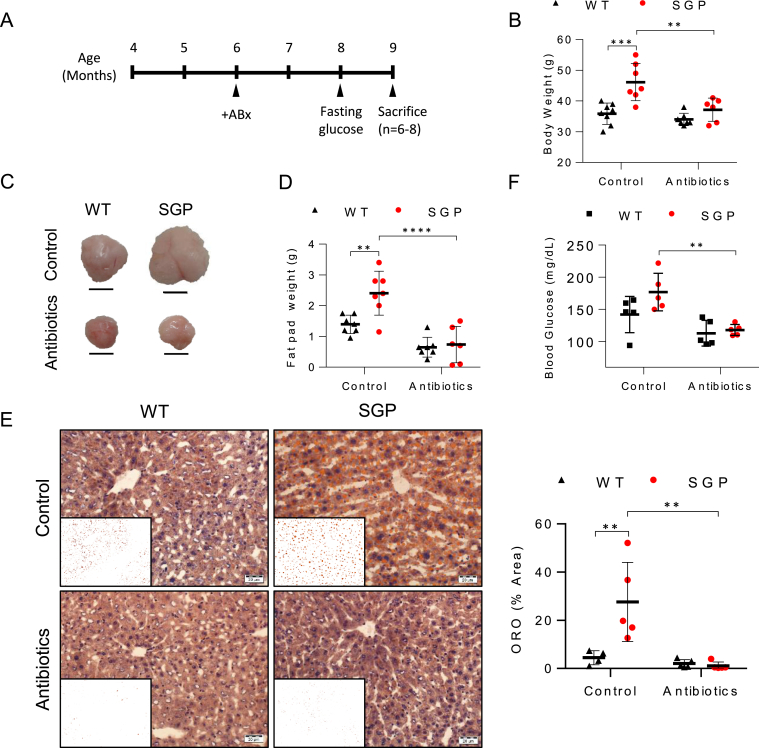
Antibiotic treatment reverses the mature-onset metabolic phenotype induced by IL-6 trans-signaling blockade. (A) Schematic representation of the experimental design. SGP and WT littermates aged 6 months were administered a NCD ± ABx in their drinking water. The mice were randomly assigned to the treatment and control groups. Fasting blood glucose levels were assessed at 8 months, and the mice were sacrificed at 9 months of age. (B) Body weights of SGP and WT littermates following NCD and ABx diets at 9 months of age (n = 6–8). (C) Photographs showing representative gonadal fat pads at 9 months of age. Scale bars, 1 cm. (D) Gonadal fat pad weights at 9 month of age (n = 6–8). (E) Microphotographs showing representative ORO-stained liver sections from 9 month old NCD or ABx-fed mice with ImageJ processed images (inset), and quantification (right) of ORO-stained areas in ImageJ processed images (n = 4). Scale bars, 20 μm. (F) Fasting blood glucose levels in control *versus* antibiotic-treated mice at 8 months (n = 5). These observations were repeated in 2 independent experiments. Numerical data are presented as mean ± SD. ∗∗*P* < .01, ∗∗∗*P* < .001, and ∗∗∗∗*P* < .0001 by 2-way ANOVA. ABx, antibiotics; NCD, normal chow diet; ORO, oil red O.

Because the age-dependent changes in microbiota-related gene expression patterns appeared to coincide with the onset of weight gain in SGP mice, we hypothesized that IL-6 trans-signaling in the liver may compensate for age-accumulated dysfunction arising from toxicities originating from the gut microbiome that would otherwise disrupt metabolic equilibrium and lead to fat accumulation. Therefore, we sought to determine whether body fat accumulation in SGP mice depends on gut microbiota. Depletion of gut microbiota by antibiotic treatment from the age of 6 months (Figure 6A) and after the onset of weight gain strikingly halted body weight gain in SGP mice, resulting in body compositions, including fat pad weight and hepatic steatosis, that were not significantly different from those of WT littermates (Figures 6B–E and S7A), without affecting food intake (Figure S7B). Antibiotic treatment also reduced fasting glucose to levels that were not significantly different from those in the WT controls (Figure 6F). Importantly, antibiotic treatment did not significantly alter the body composition of WT littermates, other than marginal reductions in fat pad weight and fasting glucose levels (*P* = ns *versus* untreated WT controls). Notably, we did not detect abnormalities or differences between WT and SGP mice in morphology of either the small or large intestines (Figure S8A and B), nor did we find differences in intestinal permeability (Figure S8C). We also detected no differences in the expression of key inflammatory cytokine mRNAs in the gut, including those of *Tnfa, IL-6*, *IL-7, IL-10, IL-22, Ifng, Ifnb, Tlr2, Tlr4, Ccl2,* and *Ccl20* (Figure S8D); thus, ruling out intestinal inflammation or changes in gut morphology as underlying factors in either the age-dependent changes in microbiota-related gene expression signatures in the liver or fat accumulation in SGP mice. Moreover, 16S ribosomal RNA gene sequencing of the fecal microbiome revealed no differences in microbiota taxonomic composition as a function of genotype or age, indicating that metabolic differences between SGP and WT mice were not associated with dysbiosis at any age (Figure S8E).

**Figure 7:**
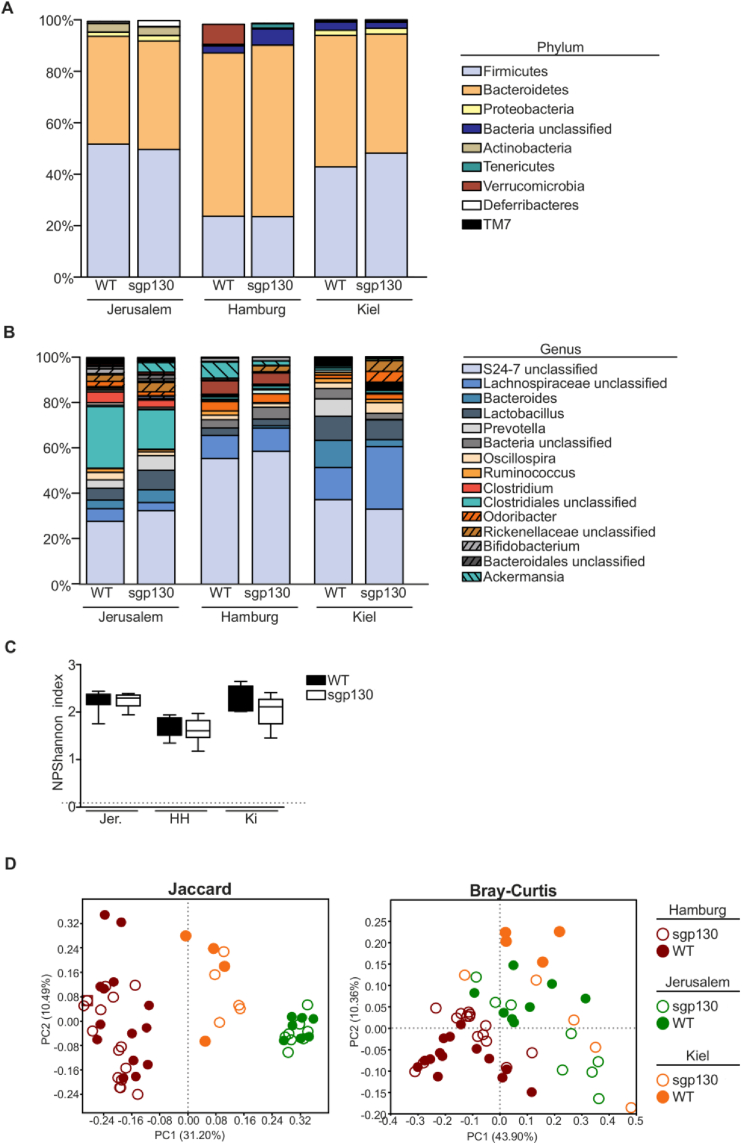
Inhibition of IL-6 trans-signaling does not alter gut microbiota composition. (A) Gut microbiota composition at the phylum level based on 16S rRNA gene sequencing of feces from mice maintained at separate facilities located in Jerusalem, Kiel, and Hamburg, and aged 8 weeks (Jerusalem, Kiel) and 11–12 weeks (Hamburg). (B) The relative bacterial abundance at the genus level did not show alterations that could be associated with the genotype in the 3 independent animal facilities. (C) Box plots of Shannon diversity indices showed that the total gut microbial diversity was independent of the mouse genotype in 3 independent animal facilities. (D) *left panel:* PCoA on Jaccard distance matrices demonstrating alterations in microbiota composition between different animal facilities. *right panel:* PCoA based on the Bray-Curtis dissimilarity index of bacterial genus abundance. PCoA, principal coordinate analysis.

Since the microbiome appeared to be integral to the mature-onset metabolic phenotype of SGP mice, we next compared the microbiomes of SGP mice and their WT littermates raised in the Jerusalem, Israel facility with mice of identical genotypes maintained in animal facilities in Hamburg and Kiel, Germany, in which SGP mice were bred but did not show the above-described metabolic phenotype. 16S ribosomal RNA gene sequencing of the fecal microbiome of the mice at the 3 animal facilities clearly showed major differences (Figure 7). This analysis indicated the presence of major differences in the gut microbiome taxonomic composition between the animal houses, which by principal coordinate analysis were highly significant (*P* = .001) (Figure 7, Table S1). The microbiome in the *SGP* colony housed in Jerusalem appeared to be enriched for Clostridiales and Clostridium populations and fewer Lachnospiraceae and Bacteroides populations than in the nonobese colonies housed elsewhere. The literature perceives Clostridiales as a harming genus in relation to obesity and metabolic syndrome, and Bacteroides and Lachnospiraceae as beneficial genera, although the benefits of the latter remain controversial.^40^, ^41^, ^42^, ^43^, ^44^, ^45^ Thus, it is plausible that the phenotypic disparity observed in geographically distant *SGP* colonies reflects differences in their gut microbiomes. We conclude that long-term IL-6 trans-signaling blockade in mice induces body fat accumulation in a manner that requires certain intestinal microbiota. However, further investigation is needed to identify the specific microbiota that support the development of the obese phenotype observed in the Jerusalem animal facility.

## Discussion

Comorbidities of metabolic syndrome are an enormous health-care burden for Western societies and are becoming increasingly common in developing countries.^46^ Aging is considered as one of the most important independent risk factors for the development of metabolic syndrome.^47^ Indeed, many of the conditions contributing to metabolic syndrome—*ie* obesity, insulin resistance, and inflammation —also increase in prevalence with age.^47^ Evidence indicates that IL-6 is an important regulator of metabolic homeostasis and contributes centrally to body weight control, particularly in obesity.^5^ Here, we investigated SGP mice, in which IL-6 trans-signaling was strongly inhibited. Our findings demonstrate that hepatic IL-6/STAT3 signaling also contributes to maintaining metabolic homeostasis in the face of an intestinal microbiome that can induce mature-onset body fat accumulation and hyperglycemia when IL-6 trans-signaling is inhibited.

A pivotal aspect of our observations is the effect of the microbiome on the production of different phenotypes of the same genotype. It is intriguing and perhaps important to note that the literature reports significant phenotypic disparities among various murine strains carrying either IL-6 or STAT3 signaling deficiencies. The metabolic consequences of IL-6 trans-signaling blockade by sgp130 observed here align with the absence of weight gain in young SGP mice reported by Kraakman *et. al.*,^30^ but are also consistent with reports of mature-onset fat accumulation in IL-6 *knockout* mice and in mice with hepatocyte-targeted STAT3 ablation.^9^^,^^10^^,^^36^ However, other studies have reported no obvious age-dependent metabolic changes in mutant mice sharing either the IL-6^−/−^ or Stat3^Δhep^ alleles identical to those in mice that do develop mature-onset obesity.^11^^,^^48^ Similarly, the obesity observed in our colony of aged SGP mice in Jerusalem was not observed in identical *SGP* colonies maintained in Hamburg and Kiel, Germany, (as well as in the Febbraio and Karin laboratories located in Melbourne, Australia, and San Diego, California, respectively), even though they were of the same origin (SRJ, personal communication). These observations suggest that subtle environmental factors may play a crucial role in the metabolic phenotypes associated with IL-6/STAT3 signaling deficiencies. Thus, our finding that body fat accumulation in SGP mice requires intestinal microbiota may be an important key to understanding these inconsistencies. This notion is supported by a recent report in which it was shown that IL33 KO mice maintained in 7 different animal vivaria manifested very different phenotypes with regard to infection by Streptococcus pneumoniae, highlighting the importance of animal vivaria in influencing the phenotype of the same genotype. In addition, in this study, the differences in phenotypes were abolished by antibiotic treatment and were therefore attributed to the observed differences in microbiome composition.^49^^,^^50^

Importantly, sgp130Fc, under the name of Olamkicept, has undergone successful phase II clinical trials in patients with inflammatory bowel disease and compassionate use in patients with very high-risk atherosclerotic cardiovascular disease. In these trials, specific inhibition of IL-6 trans-signaling did not result in adverse metabolic events.^51^, ^52^, ^53^ In contrast, the global blockade of IL-6 signaling by the neutralizing antibody tocilizumab is associated with body weight gain and increased serum lipid and cholesterol levels.^54^

Many questions regarding the phenotype of SGP mice raised in the Jerusalem animal facility remain open and warrant further study, particularly clarifying the aspects of the gut microbiome that appear to drive the phenotype in relation to aging. Determining the components of the gut microbiota and its metabolites that drive fat accumulation, identifying the sources of IL-6 in the liver, and defining the aging-related cellular processes leading to the upregulation of IL-6/STAT3 signaling are also important for delineating the mechanism underlying this phenotype.

## Conclusion

Our findings lend weight to the potential clinical significance of the reported associations of elevated sgp130 levels with obesity and diabetes in some older patients^23^, ^24^, ^25^^,^^28^ and may suggest the use of antibiotics and fecal microbiota transplantation as potential treatments for obesity, MASLD, and impaired fasting glycemia or diabetes in some adult patients.
