# Supplementary material for: Blockade of Interleukin-6 Trans-signaling in the Presence of Certain Gut Microbiota Induces Mature-onset Obesity in Mice
Source: Gastro Hep Adv. 2025 Sep 29;5(2):100819. doi: 10.1016/j.gastha.2025.100819 (PMC12681722; doi:10.1016/j.gastha.2025.100819)
Supplement: Figures S1-S8 and Table S1 [file mmc1.pdf]

Figure S1

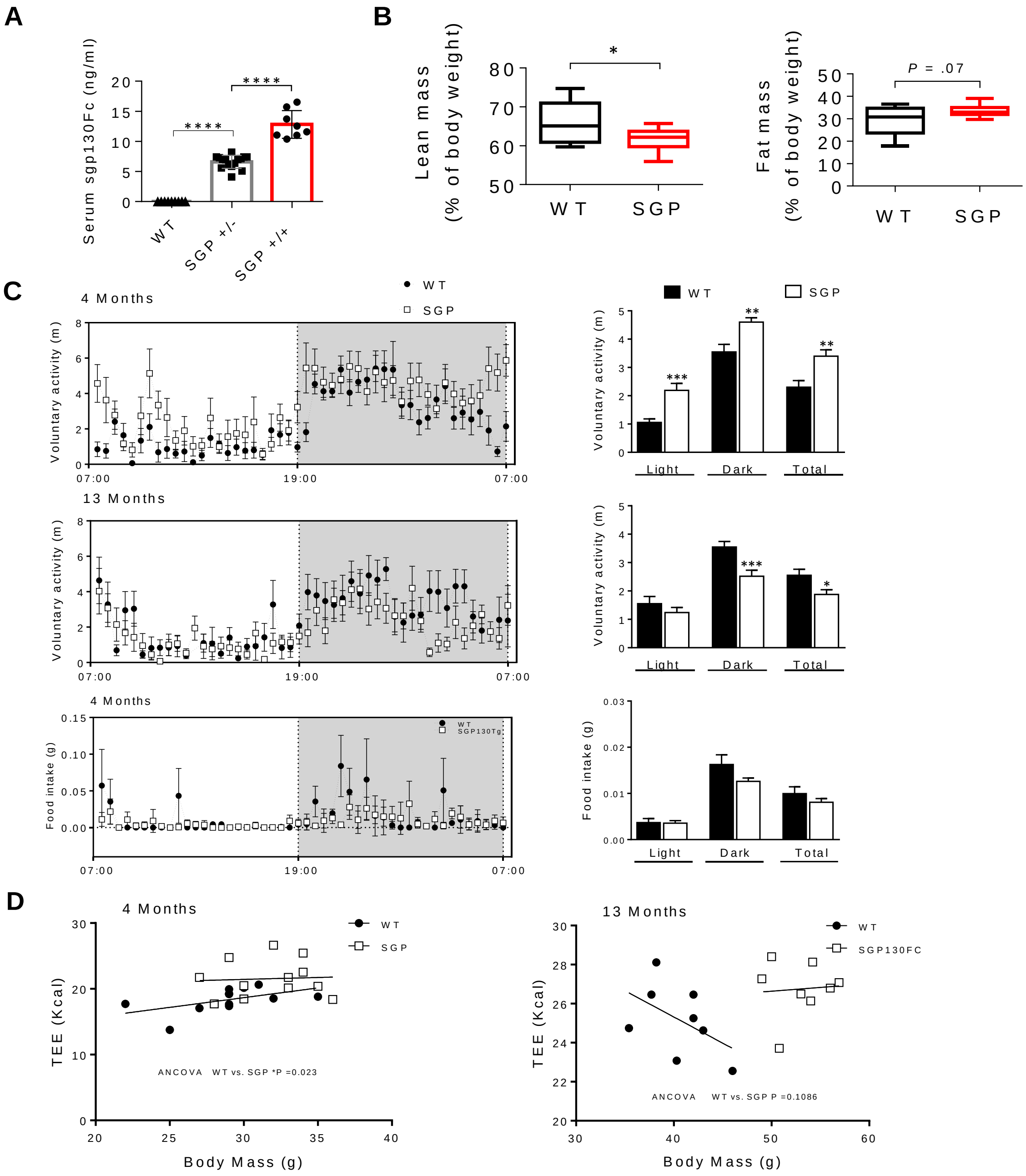

Figure S1 (Related to Figure 1) IL-6 trans-signaling blockade leads to increased fat mass and decreased lean mass with decreased physical activity at older ages.

(A) Serum concentrations of human SGP by ELISA in wild type (WT), heterozygous (-/+) and homozygous (+/+) SGP mice at 2 months of age (n=8-15).

(B) Echo MRI analysis of lean and fat body mass as a function of % body weight in SGP and WT littermates at 14 months of age (n=9-10).

(C) Voluntary activity (pedmeter) and Food Intake. Mice at 4 (n=12) and 13 (n=8) months of age were monitored by the Promethion High-Definition Behavioral Phenotyping System (Sable Instruments, Inc.) over a 24 h period. Data are mean±SEM from 4 mice per group.

(D) TEE Total vs body mass ANCOVA for WT and SGP mice at 4 and 13 months.

Data are represented as mean ± SD (A,C), or box and whiskers plots (B,D). *P < .05, and ****P < .0001 by two-tailed Student's *t* test (A,B), or by Linear regression analysis of ANCOVA (D).

Figure S2

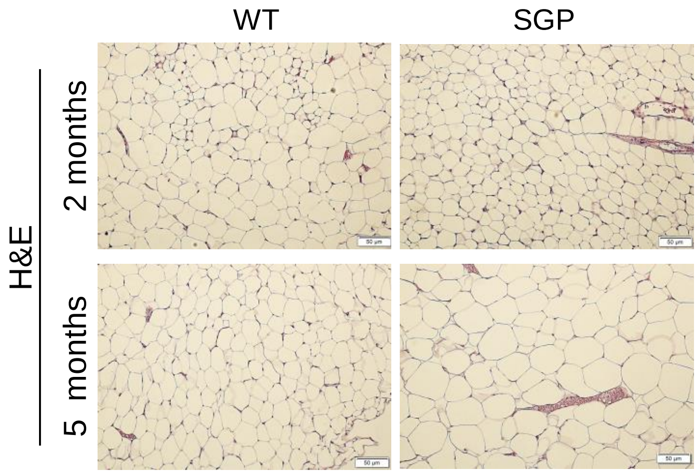

Figure S2 (Related to Figures 2) IL-6 trans-signaling blockade leads to increased fat mass, decreased lean mass, and adipocyte hypertrophy.
H&E stained sections from paraffin embedded adipose tissue from SGP and WT littermates at 2 and 5 months of age. Scale bars, 50µm.

Figure S3

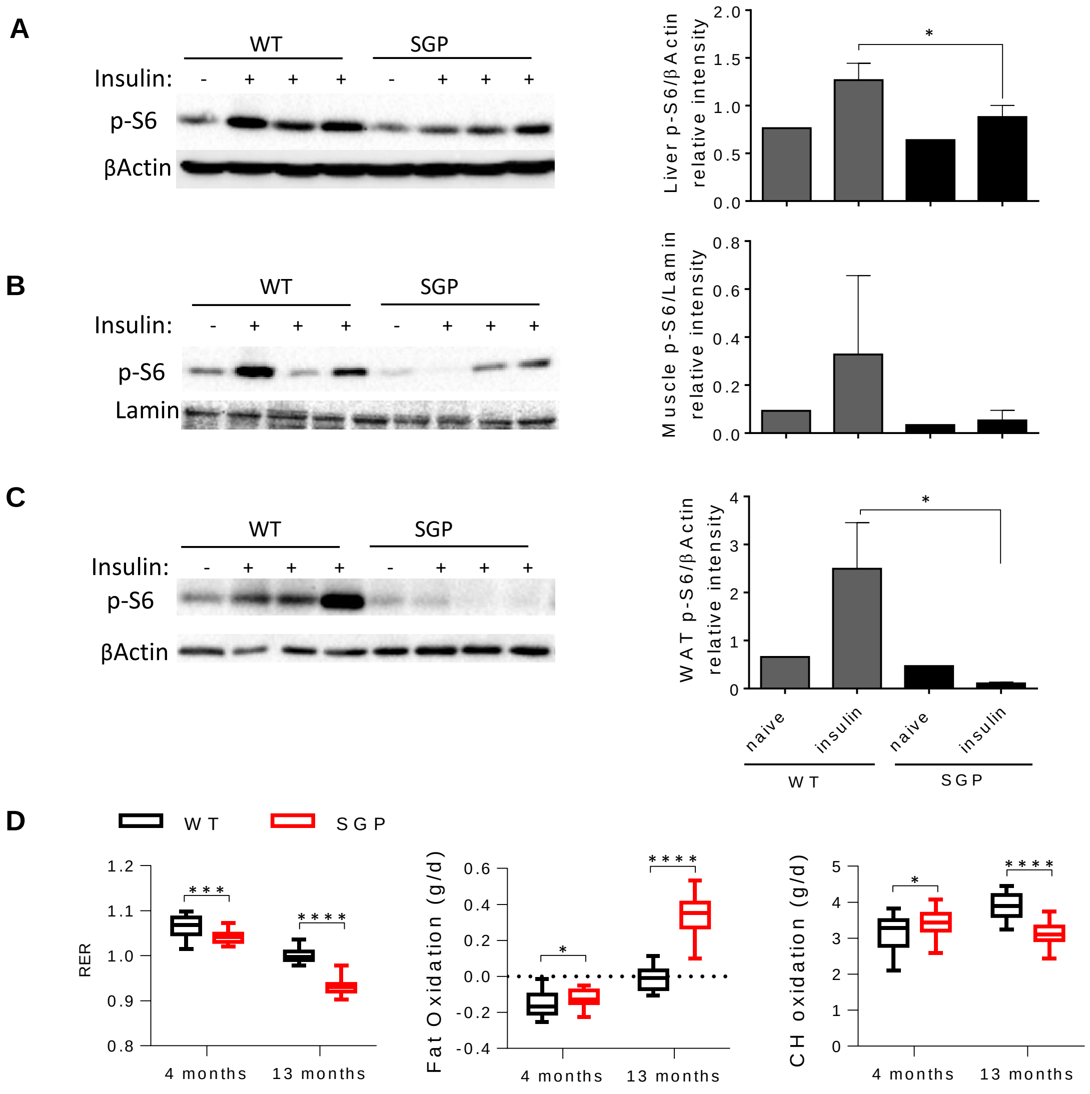

Figure S3 (Related to Figure 3). Inhibition of IL-6 trans-signaling induces glucose intolerance and peripheral insulin resistance.

(A-C) Phospho-S6 (pS6) protein levels by Western Blot analysis of liver (A), muscle (B), and white adipose tissue (WAT) (C) before and 10 minutes post insulin injection in 10 month-old SGP and WT mice. (D) Metabolic cage analysis of dark cycle metabolic activity including Respiratory Exchange Ratio (RER), fat and carbohydrate (CH) oxidation in WT and SGP mice at 4 (n=12) and 13 months (n=8).

Quantification of band intensity by ImageJ (right). (n=3 for groups of insulin-treated mice). Data are represented as mean ± SD (A-C), or box and whiskers plots (D). *P < .05, ***P < .001, and ****P < .0001 by two-tailed Student's t test.

Figure S4

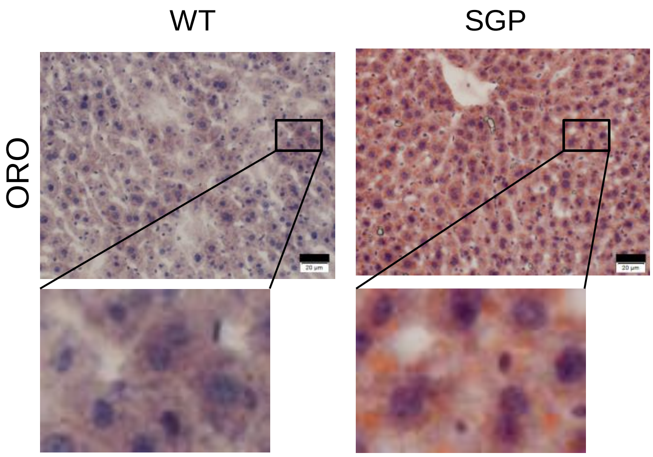

Figure S4 (Related to Figure 4). Inhibition of IL-6 trans-signaling results in steatosis at 5 months of age. ORO-stained liver sections from SGP and WT littermates at the age of 5months. Scale bars, 20 µm.

Figure S5

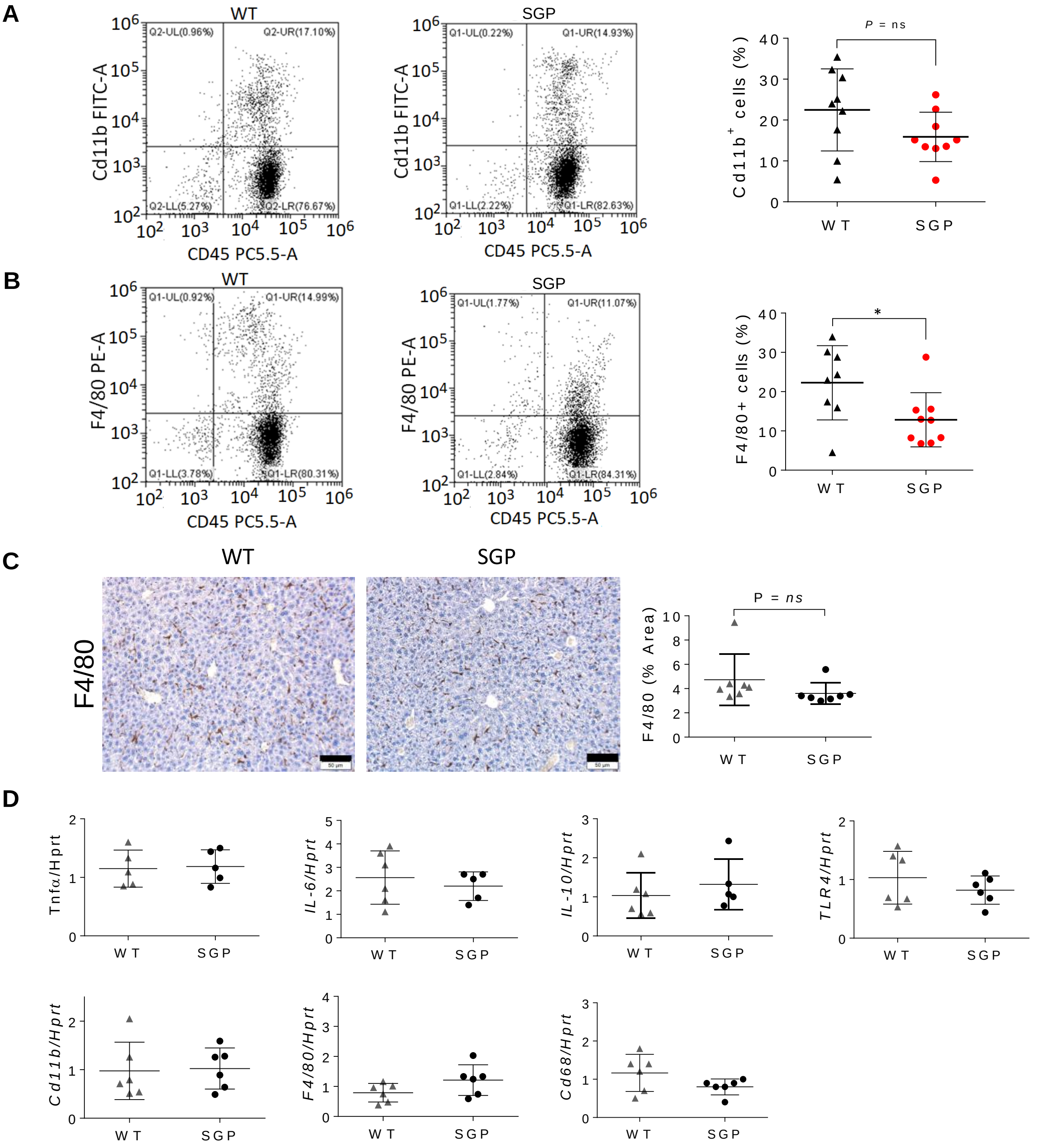

Figure S5 (Related to Figures 4). Inhibition of IL-6 trans-signaling induces steatosis without hepatic inflammation.

(A) Quantification and representative flow cytometry plots of Cd45+/Cd11b+ cells (upper right quadrants) in livers of SGP and WT littermates at 11 months of age.

(B) Quantification and representative flow cytometry plots of Cd45+/F4/80+ cells (upper right quadrants) in livers of SGP and WT littermates at 11 months of age.

(C) Representative photomicrographs of F4/80 immunostained paraffin embedded liver sections from 5-month old SGP and WT littermates and quantification (right) of F4/80+ cells as a function of area stained by ImageJ analysis, (n=8). Scale bars, 50µm.

(D) Hepatic mRNA levels by real time qPCR of indicated inflammatory and monocyte markers in SGP *versus* WT littermates at the age of 14 months.

Data are represented as mean ± SD. *$P < .05$ by two-tailed, Student's *t* test.

Figure S6

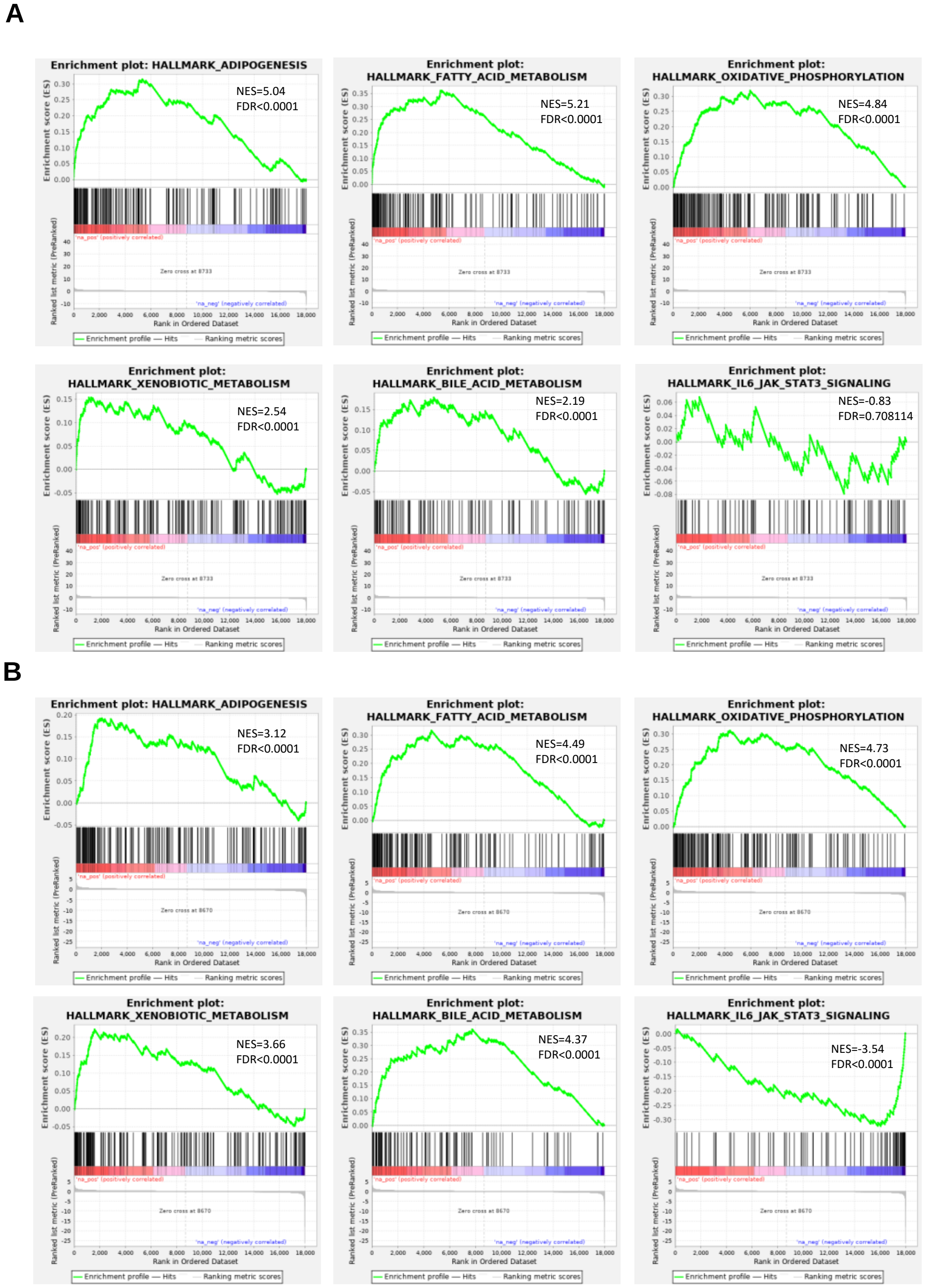

Figure S6 **(Related to Figures 5D). Transcriptome profiling in livers from WT and SGP mice.** GSEA analysis of RNAseq data of liver RNA from SGP vs WT mice aged (A) 6 months and (B) 14 months are shown. The MSigDB Hallmark gene sets related to fatty acid metabolism or metabolism of microbiome byproducts are statistically enriched (FDR < .0001) in upregulated gene sets in *SGP mice*. The IL6-JAK-STAT3 Signaling gene set is significantly enriched in the downregulated genes at 14 months, while that of 6 month is insignificant (marked with an asterisk).

Figure S7

A

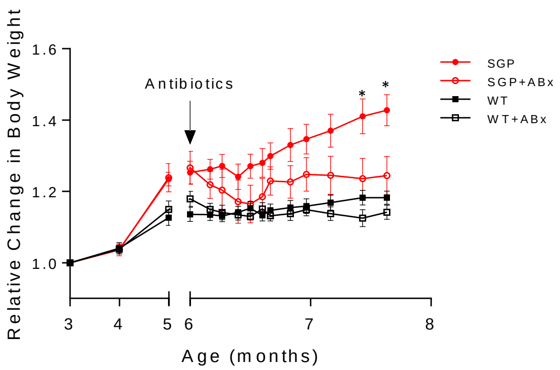

B

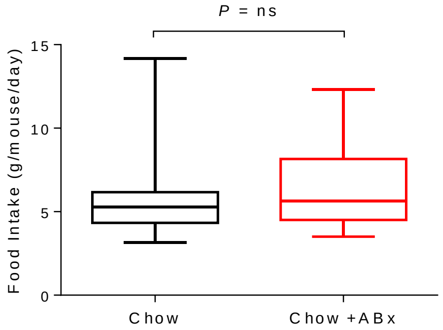

Figure S7 (Related to Figure 6). Antibiotic treatment reverses the mature-onset weight gain induced by IL-6 trans-signaling blockade without affecting food-intake. A) Relative body weight changes in SGP and WT littermates and B) Food intake by SGP mice administered a normal chow diet with or without antibiotics (ABx) in the drinking water, (n=6-8). Body weight change were calculated for each mouse relative to its weight at 3 months. Data are mean ± SEM. **P* < .05 by two-way ANOVA (A) and *P* = ns by Student's t-test (B).

Figure S8

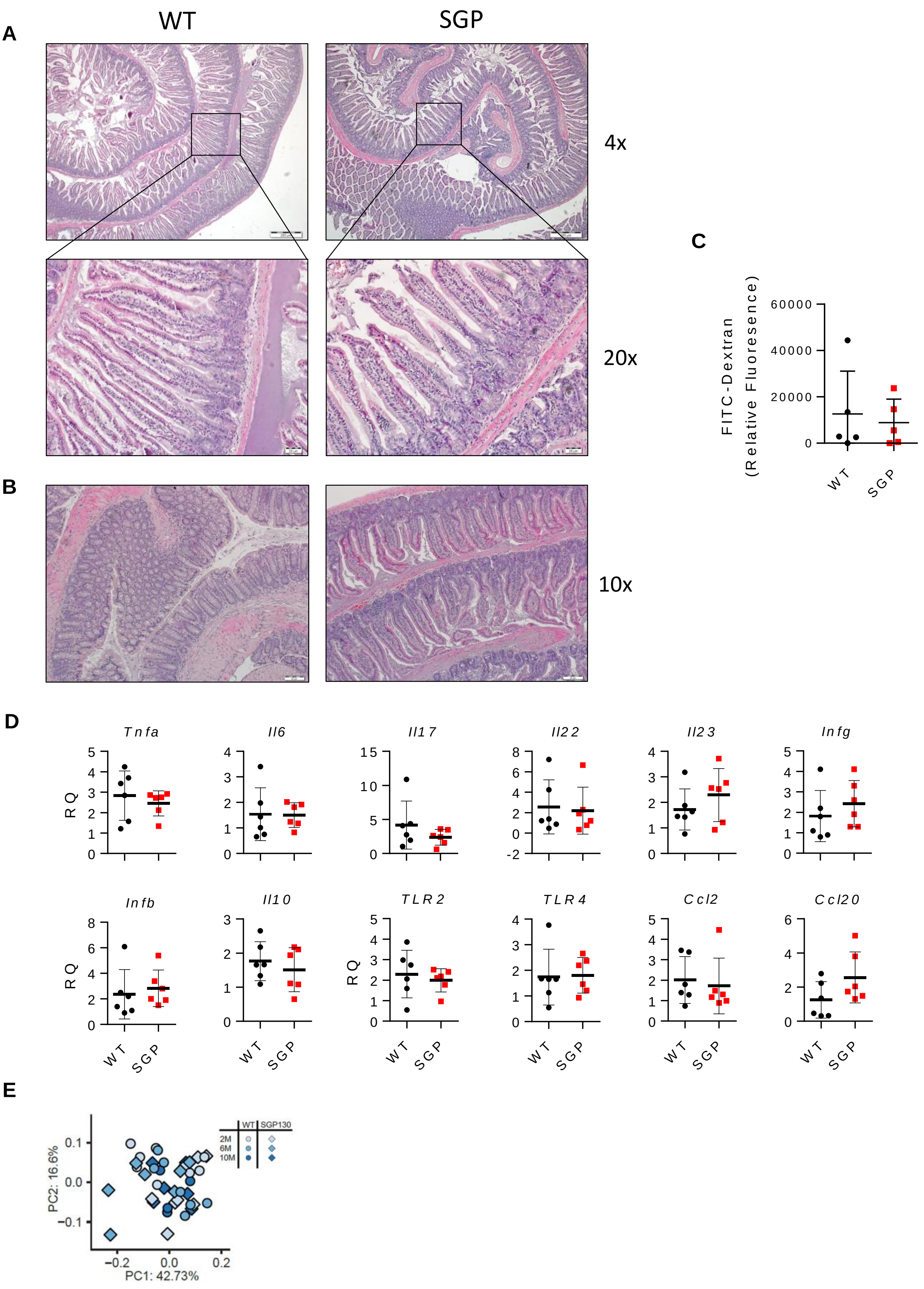

A
WT
SGP
4x
20x
B
10x
C
FITC-Dextran (Relative Fluoresence)
60000
40000
20000
0
WT
SGP
D
Tnfa
Il6
Il17
Il22
Il23
Infg
Infb
Il10
TLR2
TLR4
Ccl2
Ccl20
RQ
WT
SGP
E
PC2: 16.6%
PC1: 42.73%
WT
SGP130
2M
6M
10M

Figure S8 (continued)

Figure S8 (Related to Figure 6). IL-6 trans-signaling does not affect gut permeability, inflammation, or microbiome composition.

(A) Photographic images showing H&E of small intestinal samples from 14-month old SGP and WT littermates. Scale bars, 200µm (top) and 20µm (bottom).

(B) Photographic images showing H&E of large intestinal samples from 14-month old SGP and WT littermates. Scale bars, 50µm.

(C) Intestinal permeability indicated by serum levels of gavage-administered FITC-dextran in 10 month old mice, (n=5).

(D) Inflammatory cytokines and immune associated mRNA expression in intestinal tissue assessed by RT-qPCR.

(E) PCA analysis of taxonomic composition based on 16S ribosomal RNA gene sequencing of the fecal microbiome in WT and SGP mice aged 2, 6 and 10 months of age.

Data are represented as mean ± SD. *P* = ns by two-tailed, Mann-Whitney test.

Table SI: *P*-values are shown for permutational analysis of variance (PERMANOVA) with Bonferroni correction for multiple testing

	Jaccard			*Bray-Curtis*		
**facility**	Jerusalem	Hamburg	Kiel	Jerusalem	Hamburg	Kiel
Jerusalem		0.0003	0.0003		0.0003	0.0942
Hamburg	0.0003		0.0003	0.0003		0.0003
Kiel	0.0003	0.0003		0.0942	0.0003	
