# Supplementary material for: Blockade of Interleukin-6 Trans-signaling in the Presence of Certain Gut Microbiota Induces Mature-onset Obesity in Mice
Source: Gastro Hep Adv. 2025 Sep 29;5(2):100819. doi: 10.1016/j.gastha.2025.100819 (PMC12681722; doi:10.1016/j.gastha.2025.100819)
Supplement: Supplemental Information [file mmc2.pdf]

SUPPLEMENTAL INFORMATION

Supplemental Materials and Methods

Genetic mouse models

Sgp130Fc$^{+/+}$ (C57BL/6N) mice [1] were crossed with WT (C57BL/6J) mice purchased from Harlan Laboratories (Jerusalem, Israel) to generate heterozygous sgp130Fc$^{+/-}$ mice. Heterozygous sgp130Fc$^{+/-}$ mice were then crossed in order to generate sgp130Tg (sgp130Fc$^{+/+}$) mice and wild type (WT) (sgp130Fc$^{-/-}$) littermates. Homozygosity of the sgp130Fc$^{+/+}$ and sgp130Fc$^{-/-}$ (WT) alleles was determined by ELISA for soluble sg130Fc protein levels in the serum using anti-human sgp130 ELISA (R&D, cat.no. DY228) (Figure S1A). Male mice were used in all experiments. The primary phenotype (Figure 1) was observed in four independent experiments in male littermates, and once in both male and female non-littermates mice.

MRI Analysis

In vivo MRI analysis was performed in a blinded fashion using the M2 (Aspect Ltd, Israel), a compact, high-performance MRI system, equipped with a 35-mm mouse whole-body coil [2]. For *in vivo* MRI imaging, mice were maintained in an anesthetized state with 2% isoflurane and placed on a specially designed heated bed where physiological signals were monitored throughout the experiment to ensure the animals' wellbeing. MRI acquisition parameters include spin echo with slice thickness = 1 mm, repetition time = 450 ms, echo time = 11.5 ms, field of view = 70 mm, matrix = 256 X 256, and acquisition time = 3.5 min. Fat content was analyzed using Analyze 7.0 (Analyze Direct, USA) software.

Metabolic cage analysis

Mice were metabolically assessed in a blinded analysis by using the Promethion High-Definition Behavioral Phenotyping System (Sable Instruments, Inc., Las Vegas, NV, USA) as described previously [3]. Briefly, mice with free access to food and water were subjected to a standard 12 h light/12 h dark cycle, which consisted of a 48 h acclimation period followed by 24 h of sampling. Respiratory gases were measured by using the GA-3 gas analyzer using a pull-mode, negative-pressure system. Airflow was measured and controlled by FR-8, with a set flow rate of 2000 mL/min. Water vapor was continuously measured and its dilution effect on O2 and CO2 was mathematically compensated. The EE ANCOVA analysis done for this work was provided by the NIDDK Mouse Metabolic Phenotyping Centers (MMPC, www.mmpc.org) using their Energy Expenditure Analysis page (http://www.mmpc.org/shared/regression.aspx) and supported by grants DK076169 and DK115255. Ambulatory activity and position were monitored

simultaneously with the collection of the calorimetry data using XYZ beam arrays with a beam spacing of 0.25 cm.

Food intake

Food intake during antibiotics treatment was assessed by measurement of pre-weighed food pellets remaining after a 24-hour period. The average daily food-intake per mouse was defined as the daily average food consumption per cage measured over a two-week assessment period and divided by the number of mice per cage.

Glucose and insulin tolerance tests

Glucose tolerance (GTT) and insulin tolerance (ITT) tests were performed 2 weeks apart on fasted mice. For GTT analysis, mice received an intraperitoneal (IP) injection of dextrose (Merck) at a dosage of 1.5g/kg. Glucose levels were measured at 10, 20, 30, 40, 60, 90, and 120 minutes post-dextrose injection. ITT analysis was performed in mice by IP injection of human insulin (Actrapid) at a dosage of 0.87units/kg followed by the assessment of blood glucose levels every 20 minutes for 2 hours. Blood glucose levels were assessed using an Accu-Chek® blood glucometer and glucose test strips.

Insulin secretion

Insulin was measured in plasma samples collected from mice following overnight fasting, and 15 minutes following administration by gavage of a liquid meal consisting of Ensure Plus® supplemented with 24% (w/v) dextrose at a dextrose dosage of 2g/kg body weight. Blood samples were collected in EDTA-coated tubes (MiniCollect, Greiner Bio-one) containing protease inhibitor cocktail (Calbiochem) according to the manufacturer's instructions. Plasma samples were stored at -80º C and analyzed for insulin by Multiplex ELISA (Milliplex mouse metabolic bead panel, Millipore).

Analysis of insulin signaling in peripheral tissues

Sgp130Fc and WT littermates were fasted overnight and then sacrificed 10 minutes following injection of saline or human insulin (Actrapid) (1unit/kg, i.p.). Liver, muscle, and adipose tissue samples were snap-frozen in liquid nitrogen and stored at -80º C for protein extraction and western blot analysis.

Western blot analysis

Protein extracts were prepared from frozen tissue (~50 mg) by homogenization in lysis buffer (1% NP-40, 10 mM Tris pH 7.8, 150 mM NaCl, 40 mM EDTA, 10 mM Na-Pyrophosphate, 10 mM

NaF, 1mM PMSF, 4 mM Orthovanadate, cOmplete™, Mini Protease Inhibitor Cocktail (Merck, 11836153001), separated by polyacrylamide gel electrophoresis and subjected to Western blot analysis. For analysis of phosphorylated ribosomal protein S6 (p-S6), western blots were probed with an antibody against p-S6 ribosomal protein (Ser240/244, Cell Signaling, D68F8) followed by HRP-conjugated anti-rabbit antibody (Dako). For analysis of phosphorylated STAT3, blots were probed with anti-phosphorylated STAT3 (Santa Cruz, sc-8059), followed by HRP-conjugated anti-mouse antibody (Dako). Western blots were developed with EZ-ECL kit (Biological Industries). Blots were stripped with 0.1 M glycine pH 2.2 and re-probed with a mouse anti-β-actin antibody (Sigma, A 5316) as a loading control liver and adipose tissues, or with a rabbit anti-Lamin B1 antibody (Novusbio, NBP1-42594) as a loading control for muscle tissue. Quantification of band intensities was performed using ImageJ software.

Histology and Immunohistochemical staining

Livers, adipose, and intestinal samples were placed in 4% buffered formaldehyde for 24 hours, followed by 80% ethanol and then embedded in paraffin blocks. Tissue sections (5 µm) were deparaffinized with xylene, and hydrated through graded ethanol and stained for H&E by standard procedures. Adipocyte cell size was quantified from H&E stained thin sections using ImageJ software. Macrophages were stained using rat anti-mouse F4/80 antigen (Serotec), followed by anti-Rat HRP (Histofine) and developed with a DAB kit (Zymed). Oil red O (ORO) (Sigma) staining was performed on liver frozen sections (10µm) fixed in 0.5% Glutaraldehyde (Sigma) and counterstained with hematoxylin (Emmonya Biotech). Images of stained sections were quantified as percentage area stained positively per high power field was quantified using ImageJ software (ImageJ, RRID: SCR_003070) in 5-10 random fields per sample.

Gene Expression analysis

RNA was prepared from frozen liver samples (~50 mg) by homogenization in Trizol Reagent (Ambion) using a high-speed homogenizer (TissueLyser, Qiagen). Complementary DNA (cDNA) was synthesized from total RNA using the Quanta Biosciences qScript cDNA Synthesis Kit for mRNA, or the Quanta Biosciences qScript microRNA cDNA Synthesis Kit for miRs. RNAseq analysis was performed by the Technion Genome Center of the Israeli Institute of Technology (“Technion”, Haifa, Israel) using CEL-Seq sample preparation protocol and sequenced on an Illumina HiSeq 2500 device. RNAseq analysis was performed on mice aged 6 and 14 months (n=3) for both WT and SGP genotype. One sample each from the WT and SGP mice at 14 months appeared as outliers in the PCA analysis and were therefore excluded from further analysis, based on the recommendation of the bioinformatician. Gene expression levels were quantified by qPCR

using a Quanta Biosciences SYBR Green PCR Kit with the following primer sets and normalized to Hprt for mRNAs and Rnu6 for miRs. Gene set enrichment analysis (GSEA) was performed as described in Subramanian, *et.al.* [4].

Quantiative PCR (qPCR) of mRNA was performed using PerfeCTa SYBR Green FastMix ROX (#95073) (Quanta BioSciences Inc., Gaithersburg, MD, USA). Target mRNAs were normalized to *Hprt*. qPCR assays were performed in triplicate using an AB 7900 HT fast Real-Time PCR system (Applied Biosystems, Foster City, CA, USA) or CFX384 TM Real-Time System with C1000 Touch Thermal Cycle (BioRad, Hercules, CA, USA). The primers used for qPCR are:

Gene	Sense	Anti-Sense
Ccl2	aagccagctctctcttcctcca	gcgttaactgcatctggctga
Ccl20	ttgctttggcatgggtactg	tcggccatctgtcttgtgaa
Cd11b	gggaggacaaaaactgcctca	acaactaggatcttcgcagcat
Cd68	tgtctgatcttgctaggaccg	gagagtaacggcctttttgtga
F4/80	ccccagtgtccttacagagtg	gtgcccagagtggatgtct
Hprt	gcgatgatgaaccaggttatga	atctcgagcaagtctttcagtcct
IL-6	agttgccttcttgggactg	cagaattgccattgcacaa
IL-10	ggttgccaagccttatcgga	acctgctccactgccttgct
IL-17	ggaaagctggaccaccaca	cacacccaccagcatcttctc
IL-22	tccgaggagtcagtgctaaa	agaacgtcttccagggtgaa
IL-23	aataatgtgccccgtatccagt	gctcccctttgaagatgtcag
Infb	tccgagcagagatcttcaggaa	tgcaaccaccactcattctgag
Infg	agaggatggtttgcatctgggtca	acaacgctatgcagcttgttcgtg
Tlr4	ttcagaacttcagtggctgg	tgttagtccagagaaacttcctg
Tlr2	acaacttaccgaaacctcagac	cacacaccccagaagcatcacatg
Tnfα	gaaaagcaagcagccaacca	cggatcatgctttctgtgctc

Flow Cytometry

FACS analysis was performed on cells freshly isolated from white adipose tissue (WAT) or liver tissue samples from mice anesthetized with ketamine/xylasine following perfusion with PBS essentially as described [5] and digested with Liberase® (Roche). For isolation of the stromal vascular fraction from WAT, Liberase digestion of adipose tissue was followed by incubation for 10 minutes with 10mM EDTA, passage through a 100µm filter, and centrifugation. For hepatic tissue, Liberase digestion was followed by 70µm filtration, and centrifugation through a gradient of percoll (GE Healthcare Bio-Sciences) to separate immune cells from parenchymal cells. Following centrifugation, cells were suspended in a red blood cell lysis solution (0.155 M NH4Cl, 0.01 M KHCO3, 0.01 mM EDTA; pH 7.4) for 1 minute, followed by centrifugation and reconstitution in PBS. Fluorochrome-conjugated antibodies against the following antigens were used: anti-mouse-F4/80-PE (eBioscience), anti-mouse integrin αM-FITC (R&D), and anti-mouse cd45-PC5.5A (eBiosience).

SWATH-MS Proteomics

A detailed step-by-step protocol has been published for the sample preparation [6]. Around 30 mg of tissue from each of the 30 liver samples was manually homogenized. Proteins were extracted in 750 µL of RIPA-M buffer and then the cell pellet was lysed in 8M of urea. Both fractions were combined and quantified, then 100 µg of protein was aliquoted and precipitated in 6 volumes of 100% acetone overnight at -20°C. After centrifugation, the precipitate was resuspended in 8M urea, reduced with 12 mM dithiothreitol, and alkylated with 40 mM iodoacetamide. Samples were diluted to 1.5M urea using 0.1M ammonium bicarbonate and digested for 20 h using modified porcine trypsin at 37°C in a shaking block. The full digested samples were cleaned with C18 MACROSpin tubes (Nest Group), typically yielding around 30 – 50 µg of final cleaned peptide. For injection, 1.0 µg of each sample was loaded onto a PicoFrit emitter on an Eksigent LC system coupled to an AB Sciex 5600+ TripleTOF mass spectrometer. Data were acquired in SWATH data-independent acquisition mode (DIA) [7] (with 64 variable windows in a 120 minute gradient. For the sample analysis, a recent review also provides more detail on the full DIA pipeline [8]. In brief, the raw .wiff files were converted to mzXML using Proteowizard v2.0.1905 before being run through the OpenSWATH pipeline v2.0 [9]. The library used was taken from our prior mouse library [10]. This library contains 54511 peptides corresponding to 7106 unique protein groups. The output mzXML files for the OpenSWATH pipeline using the msproteomicstools v0.5.0 package available on GitHub. Scoring and filtering were done by PyProphet at 1% peptide FDR, followed by cross-run

alignment with TRIC using a max retention time difference of 60 seconds and a target 1% FDR, 21927 unique peptides were identified and quantified, corresponding to 2835 unique protein groups. All data were quantile normalized in R using the base "qnorm" function.

Taxonomic Microbiota Analysis

Frozen fecal samples were processed for DNA isolation using the MoBio PowerSoil kit (Quiagen) according to the manufacturer's instructions. For analysis of fecal microbiome from mice colonies maintained in the Jerusalem, the 16S rRNA gene PCR amplification, 1 ng of the purified fecal DNA was used for PCR amplification. Amplicons spanning the variable region V3/4 of the 16S rRNA gene were generated by using the following primers: Fwd 5'-GTGCCAGCMGCCGCGGTAA-3', Rev 5'- GGACTACHVGGGTWTCTAAT-3'. The reactions were subsequently pooled and cleaned (PCR clean kit, Promega), and the PCR products were then sequenced on an Illumina MiSeq with 500 bp paired-end reads. The reads were then processed using the QIIME analysis pipeline. In brief, fasta quality files and a mapping file indicating the barcode sequence corresponding to each sample were used as inputs, reads were split by samples according to the barcode, taxonomical classification was performed using the RDP-classifier, and an OTU table was created. Closed- reference OTU mapping was employed using the Greengenes database. Rarefaction was used to exclude samples with insufficient count of reads per sample. Sequences sharing 97% nucleotide sequence identity in the 16S region were binned into operational taxonomic units (97% ID OTUs).

For analysis of fecal microbiome from mice colonies maintained in the Kiel and Hamburg facilities, extracted genomic DNA was used to amplify the 16S rRNA gene specific variable region $V_{3–4}$. Success of 16 S rRNA gene amplification performance was confirmed by running an aliquot of PCR product on a 2% agarose gel. The amplicon quantities were normalized using the SequalPrep™ Normalization Plate Kit (Invitrogen), amplicons were pooled to make a library and sequenced using the MiSeq Reagent Kit v3 (Illumina) at Institute of Clinical Molecular Biology, Kiel, Germany.

Analysis of sequence reads was performed using an in-house shell script pipeline based on standard procedures for 16S rRNA gene sequence data [11]. In brief, the multiplex identifiers (MID) and 16S rRNA gene specific amplification primer sequences were removed prior to further sequence analysis. Subsequently, quality-control of sequence reads were performed as defined in Miseq SOP pipeline. Furthermore, sequences were aligned against mothur curated SILVA reference database in Mothur [12]. Sequence reads not aligned against 16S rRNA gene V3–V4 (Kiel

data sets) or V4 regions only (Jerusulam data sets) were removed from subsequent analysis. Chimeric sequences were detected by the chimera Vsearch algorithm and were also removed. In the first step, sequences were classified (threshold 80%) phylogenetically using mothur formatted greengenes (gg_13_8_99) training sets and eliminated if classified as unknown, archaea, eukaryotes, chloroplast, or mitochondria. Subsequently, reference-based (green genes) operational taxonomical units (OTUs) picking approach was implemented to cluster sequences with same phylogenetic affiliations into a phylotype at genus and phyla level (label = 1). Alpha diversity indices were calculated by Mothur. Further β-diversity estimates and Non-parametric permutational multivariate analysis of variance (NPMANOVA) was performed in PAST [13] for ascertaining the significance of clustering in sampling groups for β-diversity.

Intestinal permeability assay

Sgp130Fc mice and WT littermates aged 10 months were fasted for 5 hours during the light cycle. Mice were injected by oral gavage with 300ul FITC dextran at a concentration of 47mg/ml FITC-dextran (Sigma, Cat# 46944-500MG-F), followed by tail blood collection (~50 µl) at 4 hours-post gavage, into anti-coagulant-containing tubes (38 mM citric acid, 107 mM sodium citrate, 136 mM dextrose; 15% of collected blood weight). Bloods were kept away from light, on ice, centrifuged at 5,000 rpm for 10 minutes, and plasma supernatant was saved at -80°C. Plasma samples were diluted 1:5 in 1x PBS to give a total volume of 100 µl and transferred to a black opaque-bottom 96-well plate including a PBS blank. Relative fluorescence units were measured by a spectrophotometer at 530 nm with excitation at 485 nm. Permeability is expressed as relative fluorescence units following subtraction of PBS blank fluorescence value from all test samples.

References

1 Rabe B, Chalaris A, May U, et al. Transgenic blockade of interleukin 6 transsignaling abrogates inflammation. Blood 2008;111:1021-8.

2 Tempel-Brami C, Schiffenbauer YS, Nyska A, et al. Practical Applications of in Vivo and ex Vivo MRI in Toxicologic Pathology Using a Novel High-performance Compact MRI System. Toxicol Pathol 2015;43:633-50.

3 Udi S, Hinden L, Earley B, et al. Proximal Tubular Cannabinoid-1 Receptor Regulates Obesity-Induced CKD. J Am Soc Nephrol 2017;28:3518-32.

4 Subramanian A, Tamayo P, Mootha VK, et al. Gene set enrichment analysis: a knowledge-based approach for interpreting genome-wide expression profiles. Proc Natl Acad Sci U S A 2005;102:15545-50.

5 Cho KW, Morris DL, Lumeng CN. Flow cytometry analyses of adipose tissue macrophages. Methods Enzymol 2014;537:297-314.

6 Wu Y, Williams EG, Aebersold R. Application of SWATH Proteomics to Mouse Biology. Curr Protoc Mouse Biol 2017;7:130-43.
7 Gillet LC, Navarro P, Tate S, et al. Targeted data extraction of the MS/MS spectra generated by data-independent acquisition: a new concept for consistent and accurate proteome analysis. Mol Cell Proteomics 2012;11:O111 016717.
8 Ludwig C, Gillet L, Rosenberger G, et al. Data-independent acquisition-based SWATH-MS for quantitative proteomics: a tutorial. Mol Syst Biol 2018;14:e8126.
9 Rost HL, Rosenberger G, Navarro P, et al. OpenSWATH enables automated, targeted analysis of data-independent acquisition MS data. Nat Biotechnol 2014;32:219-23.
10 Williams EG, Wu Y, Wolski W, et al. Quantifying and Localizing the Mitochondrial Proteome Across Five Tissues in A Mouse Population. Mol Cell Proteomics 2018;17:1766-77.
11 Kozich JJ, Westcott SL, Baxter NT, et al. Development of a dual-index sequencing strategy and curation pipeline for analyzing amplicon sequence data on the MiSeq Illumina sequencing platform. Appl Environ Microbiol 2013;79:5112-20.
12 Schloss PD, Westcott SL, Ryabin T, et al. Introducing mothur: open-source, platform-independent, community-supported software for describing and comparing microbial communities. Appl Environ Microbiol 2009;75:7537-41.
13 Hammer O, Harper DAT, Ryan PD. PAST: Paleontological statistics software package for education and data analysis. Palaeontol Electron 2001;4:9.
